# Report of a Family with Craniofrontonasal Syndrome and Wolff-Parkinson-White Syndrome: Is it a New Finding?

**DOI:** 10.5935/abc.20190033

**Published:** 2019-05

**Authors:** Celal Kilit, Türkan Pasali Kilit

**Affiliations:** 1 Dumlupinar University - Faculty of Medicine - Department of Cardiology, Kütahya - Turkey; 2 Dumlupinar University - Faculty of Medicine - Department of Internal Medicine, Kütahya - Turkey

**Keywords:** Wolff-Parkinson White Syndrome, Craniofacial Abnormalities, Comparative Studies, Craniofacial Dysostose, Tachycardia, Supraventricular

## Introduction

Craniofrontonasal syndrome (CFNS; OMIM# 304110) is one of the craniofacial conditions that fall into the group called Craniofacial Dysostosis syndromes. Alternative names are Craniofrontonasal Dysplasia and Craniofrontonasal Dysostosis. CFNS is a rare X-linked disorder caused by mutations in the ephrin-B1 gene (EFNB1).^1^ CFNS predominantly affects the head, face and limbs and characterized by coronal craniosynostosis, frontal bossing, severe hypertelorism, craniofacial asymmetry, down slant palpebral fissure, broad nasal root, bifid nasal tip, grooved fingernails, curly wiry hair, and abnormalities of the thoracic skeleton.^1^ Phenotypic expression varies greatly amongst affected individuals. Paradoxical to other X-linked conditions, CFNS generally affects females more frequently and more severely than males.^1,2^ Cellular or metabolic interference due to X inactivation explains this situation. There is no accurate measurement of its birth frequency and the incidence values that were reported ranged from 1:100,000 to 1:120,000. CFNS is not diagnosed in males unless they are a member of a family known to have the condition or the father of a daughter with the condition. In females, physical characteristics play a supportive role in establishing the diagnosis but the diagnosis CFNS is determined by the presence of a mutation in the EFNB1 gene.

Wolff-Parkinson-White (WPW) syndrome is a pre-excitation syndrome which is a common cause of supraventricular tachycardia with prevalence in Western countries of 1.5 to 3.1 per 1000 persons.^3^ It is maintained by accessory pathway or pathways secondary to a developmental cardiac defect in atrioventricular electrical insulation.^3^ Among patients with the WPW syndrome, 3.4% have first degree-relatives with a pre-excitation syndrome.^4^ A familial form of WPW has infrequently been reported and is usually inherited as an autosomal dominant trait.^5-7^

There are very few cases describing association of CFNS with heart defects. We identified a CFNS family with WPW syndrome.

## Case Report

**Figure 1:**
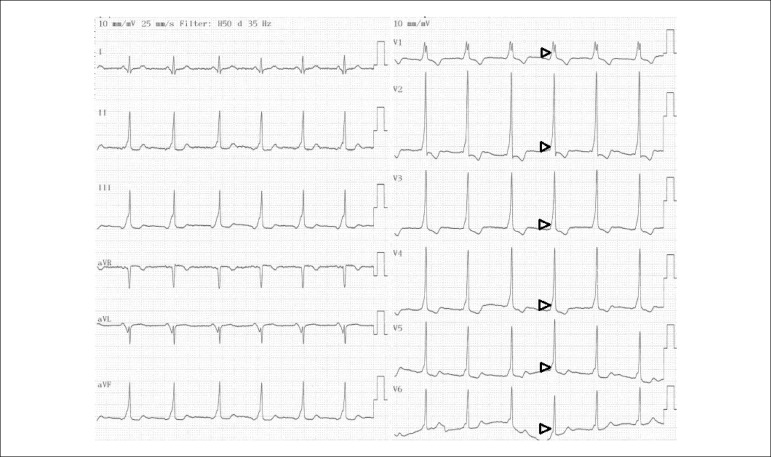
The 12-lead ECG of the patient showing Type A Wolff-Parkinson-White pattern: PR interval < 120 ms, positive delta waves (black arrowheads) in all precordial leads (V1-V6) with R/S > 1 in V1.

A 16 years old inbred girl was referred to the cardiology clinic because of paroxysmal palpitation. Her parents are consanguineous. The 12-lead electrocardiogram (ECG) showed short PR interval and Delta waves, and widened QRS complexes (Figure 1). The patient was considered as type-A WPW syndrome. Transthoracic echocardiography was normal. Patient, her sister and father have molecularly confirmed CFNS and both have heterozygous missense mutation (c.451G > A; Gly151Ser) in exon 3 of EFNB1 gene. She has undergone surgery for frontonasal dysplasia. Father was also had WPW syndrome and he had a successful catheter ablation for left lateral accessory pathway. The patient was refereed to electrophysiology department for electrophysiological study and transcatheter ablation of the accessory pathway.

## Discussion

The EFNB1 gene, which maps to Xq13.1, encodes a member of the ephrin family of transmembrane ligands for ephrin tyrosine-kinase receptor.^2^ This ephrin receptor is responsible for the cell migration, regulation of embryonic tissue-border formation, and is important for skeletal and craniofacial development.^8^ In mice, the orthologous EFNB1 gene is expressed in the frontonasal neural crest and demarcates the position of the future coronal suture. As the ephrin receptor and its EFNB1 ligand are both bound to the (trans)membrane of the cell it's cascade is activated through cell-cell interactions.^8^ These cell-cell interactions are disturbed due to the presence of cells with the mutant EFNB1 gene, as a result causing incomplete tissue-border formation.^1^

WPW syndrome is characterized with the existence of anomalous bundles of conducting tissue that bypassed all or part of the normal atrioventricular (AV) conduction system. This tissue directly connects the atria and ventricles, thereby allowing electrical activity to bypass the AV node. Tissue in the accessory pathways, which are congenital in origin and result from failure of resorption of the myocardial syncytium at the annulus fibrosis of the AV valves during fetal development, typically conducts electrical impulses more quickly than the AV node, resulting in the shorter PR interval seen on the ECG. The familial occurrence of the WPW syndrome is well documented, is typically inherited in an autosomal dominant pattern, and is sometimes associated with familial cardiomyopathy. Mutations in the genes encoding the gamma-2 regulatory subunit of adenosine monophosphate-activated protein kinase (PRKAG2) and lysosome-associated membrane protein 2 (LAMP2) have been associated with left ventricular hypertrophy in association with WPW syndrome.^4^ Studies of two families with affected subjects who had ventricular pre-excitation with conduction abnormalities and cardiac hypertrophy, mapped the PRKAG2 gene responsible for WPW to chromosome 7q34-q36.^6^ A missense mutation, Arg531Gly, was identified in affected individuals who had ventricular pre-excitation and conduction system disease with childhood onset and absence of cardiac hypertrophy.^7^

There are very few cases describing association of CFNS with heart defects such as atrial septal defect.^9,10^ To date, there are no reported cases of CFNS with WPW syndrome, suggesting that this novel finding can be part of this condition. Approximately 100 different mutations have been reported in CFNS and Gly151Ser mutation in EFNB1 gene may cause familial WPW syndrome in this CFNS family.

## Conclusion

To our knowledge, this is the first report of a family with WPW syndrome and CFNS. Genetic analyses are needed to explain this association between CFNS and WPW syndrome. Clinicians must be aware in patients with CFNS syndrome in terms of the presence of ventricular pre-excitation.
